# Antibody- and aptamer-strategies for GvHD prevention

**DOI:** 10.1111/jcmm.12416

**Published:** 2014-10-29

**Authors:** Christopher Oelkrug, Ulrich Sack, Andreas Boldt, Isis C Nascimento, Henning Ulrich, Stephan Fricke

**Affiliations:** a Fraunhofer Institute for Cell Therapy and Immunology (IZI)Leipzig, Germany; b Institute of Clinical Immunology, Universität LeipzigLeipzig, Germany; c Translational Centre for Regenerative Medicine, Universität LeipzigLeipzig, Germany; d Department of Biochemistry, Institute of Chemistry, University of São PauloSão Paulo, Brazil

**Keywords:** antibody, aptamer, GvHD, GvL, murine model, transplantation

## Abstract

Prevention of Graft-*versus*-Host-Disease (GvHD) by preserved Graft-*versus*-Leukaemia (GvL) effect is one of the major obstacles following allogeneic haematopoietic stem cell transplantation. Currently used drugs are associated with side effects and were not able to separate GvHD from the GvL-effect because of general T-cell suppression. This review focuses on murine models for GvHD and currently available treatment options involving antibodies and applications for the therapeutic use of aptamers as well as strategies for targeting immune responses by allogenic antigens.

- Introduction
- Mechanism
- Murine models
- Antibody treatment for GvHD
- Antibody treatment concerning T cells
- Antibody treatment concerning regulatory T cells
- Antibody treatment concerning B cells
- Aptamers as alternative to antibodies
- Conclusion

## Introduction

Allogeneic haematopoietic stem cell transplantation (HSCT) is an important therapy for many haematological or epithelial malignancies as well as shows promising results in non-malignant diseases [1]. The employment of donor leucocyte infusions as novel therapeutic options, non-myeloablative HSCT and cord blood transplantation have significantly helped to expand the indications for allogeneic HSCT over the last decades, especially concerning related diseases in older patients [2]. The Graft-*versus*-Host-Disease (GvHD) is still a life-threatening disease which can occur as consequence of HSCT. Van Putten *et al*. in 1967 showed that lethally irradiated mice infused with allogeneic bone marrow and spleen cells recovered from the radiation injury and bone marrow aplasia, but they died of a so-called ‘secondary disease’ [3]. The symptoms include diarrhoea, weight loss, skin changes (rough hair) and abnormal tissue regeneration within the liver [3]. This disorder was later recognized and given the name GvHD. The disease can be either considered as an acute or chronic state depending on the onset of manifestation of GvHD after HSCT. Acute GvHD occurs within 100 days after HSCT and is characterized by enteritis, dermatitis and icterus, whereas chronic GvHD occurs after this time and the symptoms can include liver damage, dry mucous (eyes, mouth, vagina), short of breath, among others. The acute GvHD (aGvHD) is responsible for nearly 15–40% of the total mortality in transplanted patients and can be seen as the major cause of mortality after allogeneic HSCT, while chronic manifestation of GvHD occurs in up to 50% of patients surviving the first weeks after HSCT [4].

Concurrently, experimentation by Billingham further detailed the occurrence of three essential elements in the development of GvHD, which was supported by their analysis [5]. First, for a GvHD manifestation to occur, the graft has to contain an immunologically competent mature T-cell population. The experimental and clinical observation of an allogeneic bone marrow transplantation (BMT) demonstrated that the severity or score of GvHD correlated with the number of transfused/administered donor T-cells [6,7]. The second observed element necessary for GvHD development indicated that the recipient (host) must be incapable of rejecting the transplanted cells. Therefore, it is necessary to suppress the host immune system through chemotherapy and/or radiation therapy prior to the stem cell administration [2]. Furthermore, as the third criteria, tissue antigens identified in the recipient were found to differ to those in the donor. As a result of these findings, further examination was necessary to provide clarification in this area of research. Consequently, studies discovered the major histocompatibility complex (MHC) [8].

The MHC includes specific proteins known as human leucocyte antigens (HLA), which are expressed on the cell surfaces of all nucleated human body cells. Analysis showed that these HLA proteins are an essential factor in the activation of allogeneic T-cells [8,9].

In addition to the three postulated causes for the GvHD, an immunological mechanism described as Graft-*versus*-Leukaemia (GvL) effect with a link to GvHD was initially observed in murine models. Here, immune cells from the donor within the graft are able to eliminate leukaemia or tumour cells remaining within the host.

## Mechanism

Graft-*versus*-Host-Disease is initiated by mature CD4^+^ and/or CD8^+^ T-cells that accompany allogeneic HSCT [10]. To prevent GvHD, all allogeneic haematopoietic stem cell transplantation patients receive immunosuppressive therapies directed against T-cells, including, in some patients, depletion of T-cells from the allograft prior to the transplantation itself. The irradiation and chemotherapy treatment applied to deplete the host immune system leads to organ damage and an additional release of pro-inflammatory cytokines (TNF-α). This also leads to a further activation of antigen-presenting cells (APCs). An increasing donor T-cell activation and release of the cytokine IL-12 by APCs, TH_1_-cells further activate CD8^+^ T-cells by the secretion of INF-γ and IL-2, which lead to an apoptosis of host cells [11].

Alloimmune T-cell responses against multiple minor histocompatibility antigens (miHAs) show an immunodominance. The presence of specific immunodominant antigens is able to predict GvHD manifestations and is also able to describe the clinical and histological phenotype. The onset of GvHD caused by MHC-mismatched transplantation results from recipient APCs that survive chemotherapy and/or radiation therapy which is beneficial in immuno-suppression. Although the manifestation of GvHD can be initiated by dendritic cells (DC) and Langerhans cells, donor-derived APCs are also essential because of a cross-presentation of recipient antigens. Both recipient- and donor-derived APCs promote T-cell-mediated GvHD. Furthermore, priming of T cells in the spleen or lymph nodes and Peyer's patches also causes GvHD. Conversely, observations show that the models undergoing lethal irradiation prior to the transplantation have no T-cell priming in Peyer's patches requirement. The phenotype of GvHD has been defined because of the influence of either activating or suppressing T-cell co-stimulatory molecules. In MHC-mismatched GvHD mediated by CD4^+^ T cells, the direct cognate interactions with recipient tissues are not necessary [11].

Different molecular mechanisms have been identified as inducers of GvHD, including the CD95–CD95 ligand, whose accumulation of CD95 ligand, observed through histological techniques, is involved in host tissue damage [12]. It was shown that perforin and granzyme pathways contribute to the GvHD manifestation and the inhibition of the perforin/granzyme pathway can be used in the treatment of GvHD [13]. Naturally occurring CD4^+^CD25^+^ regulatory T-cells in the donor or recipient can suppress the GvHD, as well as, the recipient natural killer T-cells [14].

**Fig. 1:**
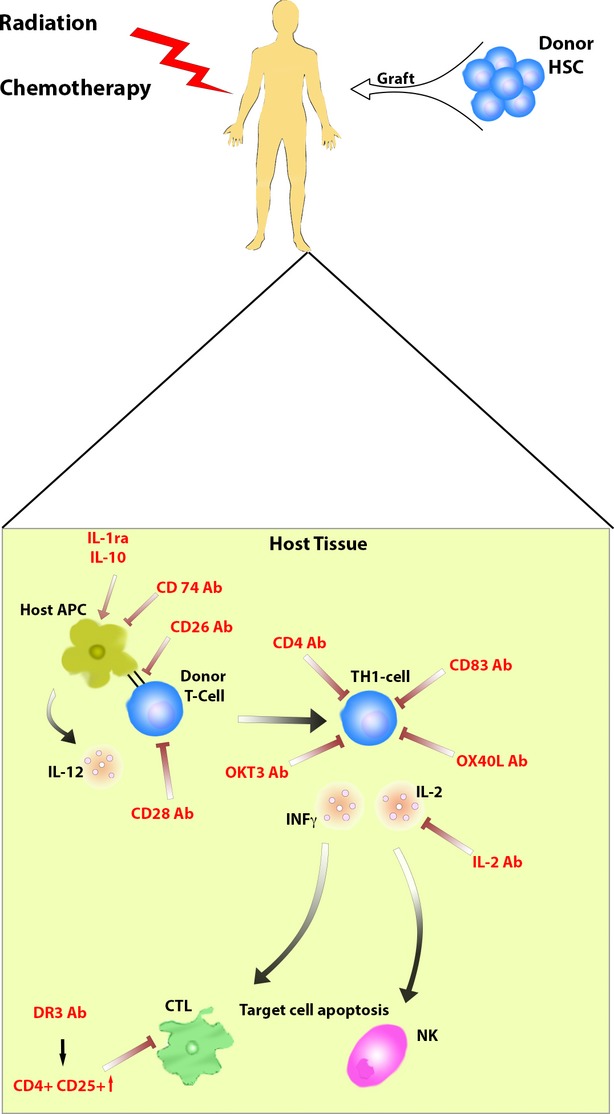
Antibody directed intervention to prevent GvHD: Antibody targets in the prevention of GvHD in host tissue after haematopoietic stem cell transplantation.

The therapeutic options include a high-dose regimen of prednisone [15] and further immuno-suppressive strategies against key elements of T-cell activation [16]. Therapies concerning OKT3® or interleukin-2 receptor antibodies [17] are seen as second line therapies to prevent GvHD. However, these strategies are associated with less long-term success, toxicity, infectious complications, relapses of the underlying haematological malignancy and a general suppression of T-cell activity. Only 50% of GvHD-affected patients respond to the current therapies. The balance between GvHD and the beneficial GvL-effect (anti-cancer capacity of donor immune cells) are crucial. Antibody directed GvHD therapies and their mode of action are shown in Figure 1.

Strategies for GvHD prevention and treatment were often not able to distinguish between different T-cell populations and therefore, not between GvHD and the GvL-effect.

## Murine models

In murine models, the onset of GvHD can be distinguished because of either MHC class I, class II, or both. Furthermore, isolated multiple miHAs are also responsible for GvHD development in these models. Although observations showed that the influence from multiple miHAs was limited relative to that induced by full MHC disparities [18]. The GvHD developing according to the response to a full MHC class I and II mismatch is dependent on specific CD4^+^ T-cells and CD8^+^ T-cells. These often result in an inflammatory cytokine storm. This massive immune reaction is capable of inducing GvHD in the target tissues without the requirement for cognate T-cell interaction with MHC on the tissue [19]. CD8^+^ T-cells trigger the development of GvHD primarily by their cytolytic function, which requires the TCR to engage MHC on the target tissue [18]. The induction of GvHD by multiple miHAs results in a process where either CD4^+^ T-cells or CD8^+^ T-cells, or both T-cell populations, depending on the specific strain combination, may play a significant role in the disease. Developing murine models addressing these mechanisms have helped to understand and refine various other mechanisms of GvHD [18]. Murine models concerning the MHC disparate and/or the miHAs disparate were able to induce specific aspects of clinically relevant GvHD characteristics.

In some cases, recipient animals undergo radiation treatment in the murine models for GvHD. Considerable care has to be taken because inbred mice have shown differing reactions to radiation. Analysis as to the maximum tolerated total body irradiation (TBI) doses for a particular strain has to be determined before experimentation to avoid organ damage. C57BL/6^wt^ mice have been shown to be more resistant than Balb/c^wt^ mice, and in addition, F_1_ hybrids are usually more resistant than the parental strain. In general, the TBI dose and its intensity play a pivotal role in the manifestation of the inflammatory expression of GvHD. Although BMT models undergoing low TBI doses and a high donor T-cell dose will result in GvHD, which is dominated by a later onset of T-cell-dependent pathology [18]. The chemotherapeutic conditioning prior to the transplantation as a myeloablative conditioning regimen with cyclophosphamide, fludarabine and busulfan (or in combination) is also used in murine models [20].

Laub *et al*. [21] developed a triple transgenic mice strain (TTG), which is a CD4 knockout C57BL/6 mice strain expressing human CD4 and HLA-DR3, as a model to study autoimmune responses. Using this TTG mice Fricke *et al*. have developed a GvHD-model in which C57BL/6 TTG mice received transplants. The host developed a severe GvHD within 45 days after transplantation. The engraftment was initially observed after 12 days associated with a GvHD development within the host animals [22]. The manifestation of the GvHD was classified according to a GvHD score and by histological analysis of the major organs. Other researchers have also described GvHD-models concerning C57Bl/6^wt^ mice as possible donors and Balb/c^wt^ as recipient mice [22]. The model established by Fricke *et al*. allows the direct investigation of antibodies against human targets (CD4) [23]. The reconstitution of white blood cells was monitored within the mice without manifestation of GvHD until the end of the experiment. In GvHD mice, however, lymphocytes and monocytes did not reach the initial levels, indicating the immuno-suppressive effect of GvH reactions which was also observed by hyposplenism in the recipient animals. For further analysis of the occurring haematopoietic chimerism, the presence of murine and human CD4 molecules, HLA-DR and H2K^b^ was examined after transplantation. Human CD4^+^, HLA-DR^+^ and H2K^b+^ cells representing either the donor or the host cells haematopoiesis were expressed from day 12 [23].

The characterization of available inbred strains as well as specific knockout or transgenic animals have helped to develop specific murine models which can be used as well for the investigation of GvH responses *in vivo*.

## Antibody treatment for GvHD

For the treatment of GvHD, immuno-suppressive strategies against key elements of T-cell reactions were already investigated in several pre- and clinical studies. For the therapy of acute GvHD, most experiences are available for OKT3® or interleukin-2 receptor antibodies and for chronic GvHD with anti-CD20 antibodies [17,23]. However, these antibodies can be associated with less long-term success and toxicity because of appearance of infectious complications. The use of monoclonal antibodies for clinical application was restricted because of the missing humanization. Antibodies of mice or other species are huge molecules with a molecular weight in the range of 150 kD that may be highly immunogenic in humans. After application of murine antihuman monoclonal antibodies, life-threatening and anaphylactic complications were observed [24]. Furthermore, the immunogenic potential of the antibodies depend on their peptide structure. IgG_4_ isotypes, for example, are less immunogenic than IgG_1_ isotypes because of the low potential for complement activation. Besides, the humanization of antibodies leads to chimeric isotypes that are less immunogenic than their originally murine counterparts. Accordingly, the investigation of alternative or improved therapeutic approaches or procedures, the treatment with antibodies or other biologicals without the need of conventional immuno-suppressive drugs are still warranted.

## Antibody treatment concerning T-cells

The membrane-bound glycoprotein CD26 (110-kD) is multifunctional and shows dipeptidyl peptidase IV enzyme activity which is present on a wide variety of cells [25]. CD26 is critical in T-cell biology, as a marker for T-cell activation. The role of CD26 in immune regulation has been extensively investigated [26]. Hatano *et al*. observed also a linkage with signalling pathways and structures involved in T-cell activation as well as APC–T cell interaction [27]. In addition, CD26 shows a co-stimulatory function in human T-cells and is up-regulated after their activation. In murine lymphocytes, CD26 is expressed in CD4^−^CD8^−^ thymocytes and its expression level is not changed by various stimulation procedures. Moreover, murine T cells are not observed to be activated *via* CD26. Therefore, for the analysis of CD26-mediated immune regulation leading to clinical applications, it was necessary to establish a GvHD-model (xenogeneic GvHD murine model) which is triggered by human T-cells. These models are generated by the transplantation of human T-cells into NOD-SCID (severe combined immuno-deficiency) mice. GvHD scores were determined by manifestation of rough hair, loss of weight and mortality after the alteration of the transplanted human T-cells into effector cells in the murine organs. The additional examination of the cytotoxic properties of human CD8^+^ T-cells after a CD26-mediated co-stimulation *in vitro*, demonstrated that the co-stimulation induced a secretion of inflammatory cytokines such as TNF-α, IFN-γ and soluble Fas Ligand, and also enhanced the expression of granzyme B. These results supported the observation that the cytotoxic function in human CD8^+^ T-cells is activated *via* the CD26-mediated co-stimulation and plays a pivotal role in the manifestation of GvHD. A possible CD26 blockade by application of a humanized anti-CD26 monoclonal antibody significantly reduced the development of GvHD. The effect of blocking CD26 was exerted by suppression of cytotoxic activity of human CD8^+^ T cells *in vivo*. The anti-CD26 antibody and the available drug, abatacept (CTLA4-Ig) showed similar results *in vivo*. In addition, an increased dosage of CTLA4-Ig showed a higher suppressive effect on GvHD but sustained suppression of engraftment of transplanted human T cells. The same dose of huCD26mAb showed no delay in engraftment. These data by Hatano *et al*. showed that CD26-mediated co-stimulatory activation in human CD8^+^ T-cells is involved in the pathogenesis of GvHD, and blocking the CD26-mediated co-stimulation resulted in prophylaxis and treatment of GvHD [27].

Yu *et al*. have investigated the role of CD28 and CTLA4 in the T-cell response to alloantigens *in vivo* by using an established GvHD-model in sub-lethally irradiated mice [28]. Experimental results indicated damage and inflammation to the recipient haematopoietic system and also bone marrow and engraftment failure, which was caused by donor T-cells. The authors showed that a monoclonal antibody directed against CD28 was more efficient than CTLA4-Ig in the prevention of GvHD. These protective effects of the anti-CD28 mAb are the result of a CD28 modulation that precludes the participation of B7:CD28 interaction in sustaining the expansion of alloreactive T cells. Another possibility might be triggered by the linkage between the monoclonal antibody directed against CD28 which results in a reduction or modulation of co-stimulatory signals by excluding CD28 from the TCR/Ag. The anti-CD28 mAb might also trigger a partial agonistic signal that causes an early termination of its clonal expansion *in vivo* [28]. An enhanced proliferation in short-term assays was observed *in vitro*. On the other hand, proliferation was prevented *in vivo*. These results recognizing nearly complete modulation of CD28 *in vivo* affected by anti-CD28 mAb were documented in the research by Yu *et al*. For further understanding of why this difference in modulation occurs, intensive investigation has to be continued. One possibility could include the interaction with Fc receptors which cause an extensive mobilization of CD28 molecules into intracellular contact caps *in vivo*.

Blocking CTLA4-signals increased GvHD, but was clearly independent from the CD28 expression on the investigated donor T cells. The CTLA4 expression and function itself were not affected by the treatment with an anti-CD28 mAb. Interestingly, in this setting CTLA4 retained its ability to decrease T-cell responses and to protect from acute GvHD manifestation in the absence of CD28. This characteristic indicates a possible cross-linking of CTLA4 that inhibits signalling events triggered through the TCR. CTLA4 might also inhibit other co-stimulatory signals or through CD134. Therefore, these results support a possible GvHD prevention through the maintaining of these CTLA4-negative regulatory signals. Moreover, observations identified that treatment with anti-CD28 mAb causes an expansion of B cells in normal mice but no effect was detected in sub-lethally irradiated dm2B6F_1_ mice.

Saito *et al*. have documented that treatment with anti-CD154 mAb improves the manifestations of GvHD induced by CD28^−/−^ T-cells [29]. The possible blockade of CD28 and CD154 in combination might be an effective strategy to induce transplantation tolerance, while preserving the CTLA4 function. In conclusion, these findings provide evidence that selective targeting of CD28 shows an immuno-suppressive behaviour in contrast to B7 and blocking CD28 and CTLA4. These results support the possibility of testing the application of anti-CD28 mAb or other selective CD28 inhibitors, which promote T-cell tolerance. The authors also recognized that CD28 inhibitors in combination with agents that block other co-stimulatory interactions such as CD154:CD40 could be a possible answer to reduce unwanted effects within the host [29].

Blazar *et al*. described the possible mechanism for the development of acute GvHD [30]. Their research suggested that co-stimulatory signals from T-cells play a pivotal role in the manifestation of acute GvHD. In addition to CD28, several other members of the TNFR family such as CD40, OX-40 and 4-1BB show potential in T-cell activation which was described as a trigger for the onset of aGvHD in humans. According to this review, these targets are currently being investigated with specific blocking antibodies to prevent a possible development of GvHD. Here, the major focus is to block the alloreactivity but not to alter the GvL-effect. Blocking the OX-40L did not show an alteration in the GvL experimental models, therefore this strategy seems promising to proceed into clinical phases [30].

Fricke *et al*. have shown that an epitope-specific *ex vivo* modulation of an allogeneic haematopoietic stem cell graft by an anti-human CD4 antibody MAX.16H5 IgG_1_ simultaneously facilitates the anti-tumour capacity of the graft (GvL) and the long-term suppression of GvHD [23]. To distinguish the GvL from GvHD effect, the anti-human CD4 antibody MAX16.H5 IgG_1_ was tested in murine GvHD and tumour models. Here, the survival rate was significantly increased in recipients receiving a MAX.16H5 IgG_1_ short-term (2 hrs) pre-incubated graft even when tumour cells were co-transplanted or when recipient mice were treated by the antibody before transplantation. It was also possible to transfer the immune tolerance from GvHD-free recipient chimaeras into third party recipient mice without the need of re-application of MAX.16H5 IgG_1_ anti-human CD4 antibodies [23].

Miwa *et al*. investigated several anti-Fas ligand inhibitory mAb also called FLIM [31]. In this study, several GvHD-models were used to understand the possible mechanism underlying the FasL blockage pathway. Here, BDF_1_ mice were lethally irradiated. Then, spleen and bone marrow cells taken from semi-allogeneic C57BL/6 mice were injected (died within 30 days). Cells from B6-gld/gld mice (lack of a functional FasL gene) survived for >80 days. A mAb (FLIM58) or Fas-Fc in these models lead to a reduction both in weight loss and mortality rate which was caused by GvHD. Interestingly, skin lesions, lymphoid hypoplasia and mononuclear cell infiltration into the liver were not improved. FLIM58 was more effective than a monoclonal antibody directed against FasL (Fas-Fc). In conclusion, FasL directed antibodies showed positive effects in the treatment of GvHD. The authors suggest that FasL might be one of the key mediators in lethal GvHD development [31].

Studies from Wang *et al*. proposed a possible clarification for antibody mediated GvHD prevention [32]. Results showed that CD83 used as a target is also known for thymic maturation and the peripheral function and longevity of CD4^+^ T-cells. Furthermore, CD83 which is also involved in B-cell maturation, peripheral B-cell function, homeostasis and DC maturation might act as a possible candidate for antibody mediated GvHD prevention.

Moreover, in a mouse model using human T-cell peripheral blood mononuclear cells (PBMCs) transplanted into SCID mice, a following treatment with anti-CD83 antibodies prevented GvHD in a dose-dependent manner and the promising results also indicated the preservation of the GvL-effect [32].

## Antibody treatment concerning regulatory T cells

Regulatory T-cells (CD4^+^CD25^+^FoxP3^+^ (T_reg_)) have been shown to decrease GvHD while preserving the GvL-effects [33]. However, their low frequency limits clinical translation. The expansion of T_reg_ *ex vivo* using cytokines (IL-2/TGFb) and antibodies (anti-CD3/anti-CD28) was investigated. Another approach for the prevention of GvHD is the development of specific strategies to expand T_reg_ *in vivo*.

The death receptor 3 (DR3) is a member of the TNF super family and its agonistic antibody (4C12) was reported to preferentially activate T_reg_ *in vivo*. Kim *et al*. have investigated the stimulation of T_reg_ through DR3 *in vivo* which resulted in an enhanced number and function of T_reg_ which was associated with less GvHD by either treating the donor mice or recipients at the time of transplant [33]. This approach of T_reg_ stimulation could serve as an alternative approach to *ex vivo* T_reg_ expansion to enhance T_reg_ function resulting in a decrease in the mortality rate and better survival with a reduced GvHD risk. In conclusion, this data show that agonistic anti-DR3 antibody stimulation can effectively activate and expand T_reg_ resulting in decreased acute GvHD in a murine GvHD-model.

## Antibody treatment concerning B cells

Milatuzumab (hLL1) is a humanized IgG_1_κ mAb that reacts with human CD74, the HLA class II-associated invariant chain [34]. Previous studies found that milatuzumab shows a potent cytotoxicity against CD74-expressing malignant B-cells *in vitro* and in xenograft models, which has lead to the ongoing clinical evaluation of milatuzumab in relapsed or refractory B-cell malignancies. Murine studies have demonstrated that milatuzumab is capable of modulating human B-cell proliferation, migration, and adhesion molecule expression, which clearly shows the therapeutic potential of this mAb in autoimmune diseases. As an HLA class II invariant chain molecule, CD74 is widely expressed in both haematopoietic and non-haematopoietic APCs, which include B-cells, monocytes, macrophages, Langerhans cells, DCs, endothelial and certain epithelial cells. Since both recipient and donor APCs, including non-haematopoietic APCs, play critical roles in the initiation of GvHD, milatuzumab might have therapeutic potential for GvHD by altering recipient and/or donor APCs. It inhibits allogeneic T-cell proliferation in specific leucocyte reactions. In a developed human/mouse xenogeneic SCID mouse model in which GvHD is induced and mediated by transplantation of human CD4^+^ T-cells and DCs, milatuzumab effectively prevents the manifestations of acute GvHD. It is also able to suppress the serum levels of secreted human IFN-γ and IL-5, and also decreases the infiltration of human lymphocytes into GvHD target organs (lung, liver, and spleen). The therapy with milatuzumab significantly promotes survival (90% *versus* 20%) and does not affect the number of cytomegalovirus-specific, IFN-γ–producing human CD8^+^ T-cells in allogeneic mixed leucocyte reactions [34]. The anti-GvHD potential of an anti-CD74 mAb is also supported by observations that macrophage specific migration of the inhibitory factor, and of CD74 ligand, is involved in the development of acute GvHD in a murine model of allogeneic stem cell transplantation [34].

The prevention of GvHD with antibodies directed against specific targets mentioned in this review are also summarized in Table 1 to show the mechanism triggered by these antibodies and displaying the used murine GvHD-models to investigate their potential in *in vivo* studies.

**Table 1:** Overview of murine GvHD models and treatment strategy

Cell type	Target	Description	GvHD-Model	Results	Reference
T-cells	CD26	Membrane-bound glycoprotein dipeptidyl peptidase IV enzyme; T-cell activation and APC–T cell interaction	x-GvHD (human T-cells)	Anti-CD26 mAb; activity of CD8↓	Hatano *et al*. [27]
T-cells	CD28	Expressed on T-cells as co-stimulatory signal, required for T-cell activation. CD28 is the receptor for CD80 (B7.1) and CD86 (B7.2)	Sub-lethally irradiated (B6.Ly5.1 × bm12)F_1_ mice transplanted with purified CD4 cells from B6.Ly5.2 donors	Anti-CD28 mAb, inhibition of T-cell proliferation, induction of T-cell tolerance	Yu *et al*. [28]
T-cells	OX-40L	Ligand for CD134; expressed on DC2s enabling amplification of Th_2_ cell differentiation	MHC class II (bm12) or class I (bm1) recipients; purified LN CD4^+^ or CD8^+^ T-cells from B6, B6 OX40^−/−^, 129/Sv OX40L^−/−^	Blockage of alloreactivity, no alteration of the GvL-effect	Blazar *et al*. [30]
T-cells	CD4	Glycoprotein found on the surface of immune cells such as T helper cells, monocytes, macrophages and dendritic cells	BALB/c (irradiated), administration of spleen and bone marrow cells of TTG mice	Increased survival rate after pre-incubation with the antibody, GvL-effect was not altered	Fricke *et al*. [23]
T-cells	Fas L	Type-II transmembrane protein belonging to the tumour necrosis factor (TNF) family	BDF_1_ (irradiated), administration of spleen and bone marrow cells of C57BL/6, models with B6-gld/gld (lack of functional FasL gene) survived	FLIM58 antibody more effective than a monoclonal Ab against FasL; FasL directed antibodies showed positive effects in survival	Miwa *et al*. [31]
T-cells	CD83	Thymic maturation and peripheral function and longevity of CD4^+^ T-cells	x-GvHD, human PBMCs in SCID mice	Prevention of GvHD in a dose-dependent manner and preservation of GvL-effect	Wang *et al*. [32]
T_reg_	DR3	Member of the TNF family	C57BL/6 → (C57BL/6 × DBA/2) F1	Enhanced number and function of T_reg_ *in vivo*	Kim *et al*. [33]
B-cells	CD74	Expressed on haematopoietic a non-haematopoietic antigen-presenting cells	x-GvHD, human CD4 T-cells and dendritic cells	Inhibits allogeneic T-cell proliferation, suppresses IFN-γ and IL-5, inhibits T-cell infiltration	Chen *et al*. [34]

## Aptamers as alternative to antibodies

Aptamers are single-stranded DNA or RNA molecules, developed by an *in vitro* selection process of a combinatorial oligonucleotide pool against a target molecule of biological or therapeutic interest, by using the SELEX (systematic evolution of ligands by exponential enrichment) technique. Exposure of target molecules to the oligonucleotide library is followed by elution of target-binders and amplification of those by PCR. Reiterative SELEX cycles are performed until the initial combinatorial pool containing of up to 10^15^ different sequences has been purified to a homogeneous fraction of target-binders. Aptamers are identified from this pool by cloning and DNA sequencing is further classified by structural conserved motifs in their previous random regions and binding affinity and selectivity [35]. Applications of aptamers for *in vivo* applications and as therapeutic agents are promising, as chemical modifications are routinely introduced providing stability to nuclease attack and largely prolonging aptamer half-life in the plasma.

Aptamers rival monoclonal antibodies in many applications, including diagnosis and therapy. Moreover, aptamer-target interactions reveal low dissociation constants, ranging from nanomolar to femtomolar, similar to those between monoclonal antibodies and antigens.

Unlike monoclonal antibodies which have high immunogenicity, high cost of production, lot-to-lot variation and are sensitive to elevated temperature, aptamers are produced *in vitro* and no organisms are required. There is no variation in the synthesis of different batches, they are thermostable, have low production costs and present low or no immunogenicity. Aptamers are flexible molecules and being capable to bind to hidden epitopes, which cannot be reached by antibodies [36]. A variety of aptamers has been developed, which antagonize protein dysfunctions involved in disease development and have been tested in clinical trials [37–40], including macular eye degeneration and choroidal neovascularization, thrombus formation in coronary artery disease, von Willebrand's disease, non-small lung tumour and renal cell carcinoma [41].

Aptamers have also been developed for interfering with immune responses, for instance by binding and inactivation of interleukin-6 or TNF-α [42,43]. Autoimmune diseases have also been targeted, including autoimmune antibodies in myasthenia gravis, midkine in experimental autoimmune encephalomyelitis, IL-17A signalling factors in glucose-6-phosphate isomerase-induced rheumatoid arthritis and myelin oligodendrocyte glycoprotein -induced experimental autoimmune encephalomyelitis [44,45]. Efforts have been undertaken for developing aptamers targeting control mechanisms of protective immunity for different applications, including anti-cancer cell immunity and protection against septic shock. As an example, aptamers mediated siRNA inhibition of the mTOR complex 1 (mTORC1) function in CD8+ T-cells augmented their differentiation into memory T-cells potentiating anti-tumour immunity [46], while CpG oligonucleotide aptamers through TLR-9 activation led to anti-inflammatory responses [47].

Aptamers developed against the CD4 antigen inhibiting CD4^+^ T-lymphocyte function, provided evidence that T lymphocyte-mediated immune response can be successfully targeted by using the SELEX technology [48]. Further aptamers with therapeutic interest in regulation of immune responses were developed as blockers of IFN-γ-receptor binding and inhibitors of human non-panreatic phospolipase A2 [49,50]. Aptamer-targeting CD28 reduced the binding of B7.2-Fc, expressed on APC, turning the lymphocyte into an anergic state [51]. CD28 on T-cells activated by B7.1 and B7.2 expressed by APC and together forms the major co-stimulatory system involved in activation and expansion of T-cells. Therefore, it has been suggested that CD28 can participate at GvHD development, and several studies have shown modulation of CD28-B7 interaction as a promising therapy to prevent GvHD [52–54]. Recently, an aptamer developed as high-affinity ligand of CD8 to inhibit activation of cytotoxic T lymphocytes was used as therapeutic strategy to use in patients with GvHD [55]. Having in mind internalization of the CD8 surface receptor, an anti-CD8 aptamer-siRNA chimera delivery system was developed for knocking down GNLY gene expression and inhibiting alloreactive response. The authors of this study conclude that aptamer-coupled RNAi constructs provide cutting edge technology for new therapeutics targeting GNLY- and CD8-expressing cells.

**Fig. 2:**
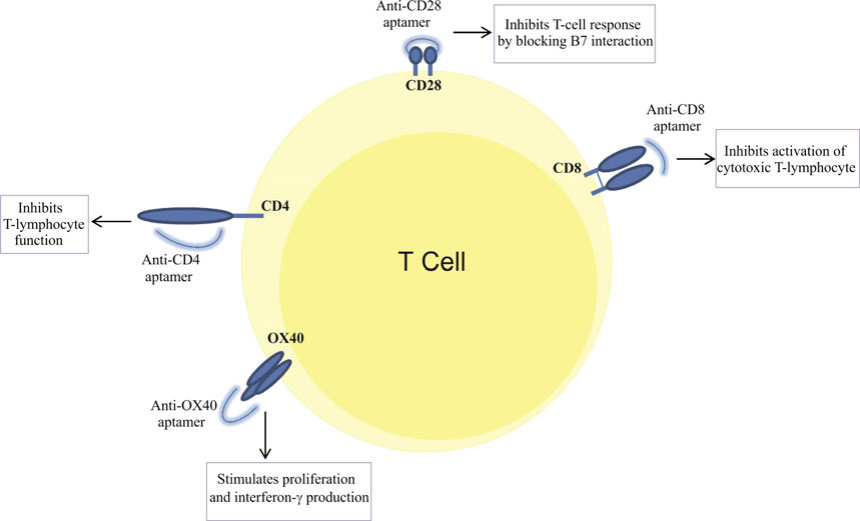
Interference of aptamers with T-cell activation: Aptamer targets (CD28, CD8, CD4 and OX40) concerning T-cell activation in the prevention of GvHD after stem cell transplantation.

Moreover, an aptamer that targets OX40 on activated T-cells which stimulates cell proliferation and interferon-γ production was developed. The authors suggest that an engineered form of this aptamer able to block OX40-OX40L interaction may be a promise for the treatment of immune-mediated disease such as GvHD [56]. The interference of aptamers with T-cell activation is illustrated in Figure 2.

Innate immunity pathways, such as for protection against viral infection, involves stimulation of the TLR receptor pathway or the RNA helicase RIG-I, leading to down-stream signalling and activation of transcription factors NF-κ B, IRF3 and IRF7 [57]. NF-κ- signalling has been targeted by intracellular-acting aptamers for regulation of alternative splicing and exon exclusion. This innovative strategy aims at rewiring pathways for changing cellular responses, connecting disease markers to non-invasive sensing and reprogrammed cellular fates [58].

Aptamers inhibiting cytotoxic T lymphocyte activation involved in immune disorders are promising tools for evaluation in clinical trials. Previous conducted phase I and II clinical trials have evaluated the use of aptamers in therapy [41]. The problem of degradation has been efficiently solved by chemical modifications of the aptamers. The stability of DNA aptamers can be readily augmented by inverting the nucleotide at the 3′-terminus, protecting them against 3′ exonuclease activity. RNA pools for *in vitro* selection are protected against nuclease activity by the incorporation of 2′F- or 2′-NH_2_-pyrimidines [59]. For therapeutic application, aptamers are then further scanned for site-specific substitution of unmodified and 2′F- or 2′-NH_2_-pyrimidines by 2′-O-methyl-nucleotides, as shown successfully for the FDA-approved anti-VEGF165 aptamer [39,60].

Rapid renal clearance and accumulation of aptamers in tissues following repeated applications require further attention. Pharmacokinetics of aptamers can be improved by coupling them to high molecular weight moieties such as polyethylene glycol or cholesterol, aiming to resolve the problem of tissue accumulation following repeated aptamer treatment and to prevent aptamers from forming basophilic granules in the cytoplasm of renal proximal tubular epithelial and reticuloendothelial cells and in the liver [41]. Improved aptamers have been developed into the clinics, such as the FDA-approved Macugen for the treatment of macular eye degeneration [40]. The advantages of aptamers compared to antibodies in easy target access, similar to small molecule drugs and resistance to degradation will widen their therapeutic applications, including in GvHD and other immune system-related disorders.

## Conclusion

Graft-*versus*-Host-Disease is still considered as a life-threatening disease after allogeneic HSCT and classified into an acute and chronic form based on the time of onset [61,62]. Bone marrow [63], peripheral mobilized stem cells [64] and umbilical cord blood [65] are the common sources for HSCT. Clinical studies showed that 30–40% of patients develop a moderate form of GvHD, but 10% develop a severe one, which is difficult to control [66–68]. T cells play a pivotal role in the development of GvHD. Subsequent tissue damage severely impairs organ function, especially in gut, skin, liver and eyes [69]. Current therapeutic options are still limited and lead to a suppression of the entire immune system which enhances the possibility for infections or development of malignant tumours. The most widely used GvHD prophylaxis following patient conditioning includes a combination of a calcineurin inhibitor (*e.g*. cyclosporine) with methotrexate. Other regimens for GvHD prophylaxis include antibodies directed against the anti-tumour necrosis factor and manipulation of the graft such as T-cell depletion. First-line therapy options include high-dose prednisone [15]. Only 50% of GvHD-affected individuals respond to the current therapy. Often, there are various side effects, infectious complications and relapses of the underlying haematological malignancy. Furthermore, the balance between GvHD and the beneficial GvL-effect (anti-cancer capacity of donor immune cells) is highly important for the reconstitution of a healthy immune system of patients suffering from haematological malignancies [70]. Studies with murine models have clearly contributed to the understanding the biological mechanism of GvHD development after stem cell transplantation as well as comprehension of cellular interactions and their impact on GvHD. Some publications state that observations made in murine models for GvHD do not correspond to an acute GvHD but to a chronic type of this disease. Others describe this as a GvHD-like manifestation [71,72].

Further improvements and more powerful tools to analyse the existing murine GvHD-models will undoubtedly provide explanations for better comprehension of this complex immunological process in the future. Of particular importance is the identification of therapeutic options including novel antibodies and aptamers to prevent or cure GvHD.
